# Oxidative stress, redox status and surfactant metabolism in mechanically ventilated patients receiving different approaches to oxygen therapy (MecROX): An observational study protocol for mechanistic evaluation

**DOI:** 10.3310/nihropenres.13567.1

**Published:** 2024-04-26

**Authors:** Ahilanandan Dushianthan, Daniel Martin, Paul Mouncey, Tasnin Shahid, Lamprini Lampro, Amelia Francis Johnson, Victoria Goss, Angelica Cazley, William Herbert, William Jones, Mark Lamond, Florence Neyroud, Karen Salmon, Julian Lentaigne, Magdalena Minnion, Madhuri Panchal, Grielof Koster, Helen Moyses, Anthony D Postle, Martin Feelisch, Michael P W Grocott

**Affiliations:** 1 General Intensive Care Unit, University Hospital Southampton, Southamnpton, Hampshire, SO16 6YD, UK; 2 NIHR Biomedical Research Centre, University Hospital Southampton, Southampton, Hampshire, SO16 6YD, UK; 3 Faculty of Medicine, University of Southampton, Southampton, England, SO16 6YD, UK; 4 Peninsula Medical School, University of Plymouth, Plymouth, England, PL6 8BT, UK; 5 Department of Intensive Care, University Hospital Plymouth, Plymouth, Devon, PL6 8DH, UK; 6 Intensive Care National Audit and Research Centre, London, England, UK; 7 Clinical Trials Unit (CTU), University Hospital Southampton, Southampton, Hampshire, SO16 6YD, UK; 8 Patient and Public Involvement Team, University Hospital Southampton, Southampton, Hampshire, SO16 6YD, UK

**Keywords:** Oxygen, Hyperoxia, Mechanical ventilation, Surfactant, Redox, Oxidative stress

## Abstract

**Background:**

MecROX is a mechanistic sub-study of the UK-ROX trial which was designed to evaluate the clinical and cost-effectiveness of a conservative approach to oxygen therapy for invasively ventilated adults in intensive care. This is based on the scientific rationale that excess oxygen is harmful. Epithelial cell damage with alveolar surfactant deficiency is characteristic of hyperoxic acute lung injury. Additionally, hyperoxaemia (excess blood oxygen levels) may exacerbate whole-body oxidative stress leading to cell death, autophagy, mitochondrial dysfunction, bioenergetic failure and multi-organ failure resulting in poor clinical outcomes. However, there is a lack of *in-vivo* human models evaluating the mechanisms that underpin oxygen-induced organ damage in mechanically ventilated patients.

**Aim:**

The aim of the MecROX mechanistic sub-study is to assess lung surfactant composition and global systemic redox status to provide a mechanistic and complementary scientific rationale to the UK-ROX trial findings. The objectives are to quantify *in-vivo* surfactant composition, synthesis, and metabolism with markers of oxidative stress and systemic redox disequilibrium (as evidenced by alterations in the ‘reactive species interactome’) to differentiate between groups of conservative and usual oxygen targets.

**Methods and design:**

After randomisation into the UK-ROX trial, 100 adult participants (50 in the conservative and 50 in usual care group) will be recruited at two trial sites. Blood and endotracheal samples will be taken at 0, 48 and 72 hours following an infusion of 3 mg/kg *methyl*-D _9_-choline chloride. This is a non-radioactive, stable isotope of choline (vitamin), which has been extensively used to study surfactant phospholipid kinetics in humans. This study will mechanistically evaluate the *in-vivo* surfactant synthesis and breakdown (by hydrolysis and oxidation), oxidative stress and redox disequilibrium from sequential plasma and bronchial samples using an array of analytical platforms. We will compare conservative and usual oxygenation groups according to the amount of oxygen administered.

Trial registration: ISRCTN

ISRCTN61929838, 27/03/2023 https://doi.org/10.1186/ISRCTN61929838.

## Plain Language Summary

Although oxygen is necessary, excess oxygen can be harmful especially to those requiring artificial ventilation. A national research study (UK-ROX) is looking to find out whether giving less oxygen to patients in ICU will improve their survival compared to standard care (more oxygen). The UK-ROX trial will not be able to assess how exactly excess oxygen may cause harm. Therefore, this study will run in parallel with UK-ROX to look in more detail at how excess oxygen might affect the lungs. Usually, lungs are kept open by a detergent-like material within them called "surfactant" so they can function properly. Too much oxygen can kill the cells that make surfactant or increase the breakdown of surfactant. Too much oxygen can also cause powerful chemical reactions (called oxidative stress) that can damage cells all around the body, causing organs to malfunction and worsen the patient’s condition.

One hundred patients from the UK-ROX trial will be recruited to determine whether surfactant and oxidative stress play a role in oxygen-induced lung damage. We will take blood and lung fluid samples from these participants three times during the study. Patients on a breathing machine will usually receive sedation as part of their treatment, so they are unlikely to be able to consent to participate in the study. So, we will approach a nominated person (usually their next of kin) for their opinion on whether they think the patient would want to participate. We will ask patients for their consent at the earliest opportunity when they regain consciousness.  Patient and public members helped design this study. Results will be disseminated through media, health charities, publications, and scientific conferences. We will provide evidence for any oxygen-related guidelines for doctors and nurses to provide the best possible care for ICU patients needing oxygen therapy.

## Introduction

Oxygen therapy is the most commonly used medical intervention in the intensive care unit (ICU). Most mechanically ventilated patients require supplementary oxygen, yet the optimal therapeutic oxygen levels are not known. The UK-ROX randomised controlled trial is an NIHR HTA funded study that aims to evaluate the clinical and cost-effectiveness of a conservative approach to oxygen therapy to achieve a low oxygen saturation target [SpO ^2^ 90±2%] compared with standard therapy, determined by local practices in mechanically ventilated patients ^1^ (Trial registration ISRCTN 13384956, 08/12/2020, https://doi.org/10.1186/ISRCTN13384956). The primary aim of this study (MecROX) is to provide a mechanistic evaluation of systemic, alveolar redox status and dynamic surfactant biology following different oxygen therapeutic strategies in mechanically ventilated patients enrolled into the UK-ROX study. This is an observational sub-study embedded within the UK-ROX and all patients will be co-enrolled with UK-ROX interventional study.

Hyperoxia has been used to induce acute lung injury in animal models. Following exposure, animals develop significant alveolar cellular damage with capillary leak, resulting in pulmonary oedema and development of acute respiratory distress syndrome ^2– 4^. Hyperoxic challenge studies of humans are limited. High concentrations of inspired oxygen for short periods in healthy humans can lead to substernal distress, pleuritic chest pain, cough, progressive dyspnoea, decline in vital capacity and carbon monoxide diffusion (DLCO) capacity and abnormalities of tracheal mucociliary movement ^5– 7^. Moreover, inspired oxygen of >95% for 17 hours can lead to significant alveolar-capillary leak with increased fibroblast recruitment and proliferation ^8^. These limited human and animal studies, support the notion that oxygen has the potential to cause acute lung injury and lethality in a normal uninjured lung ^3, 9^. However, more importantly, an injured lung may respond differently to hyperoxic challenges than a normal lung. The implications of combined insults such as a primary lung pathology, critical illness, and mechanical ventilation in combination with oxygen toxicity in the development and progression of acute lung injury are largely unknown.

### Pulmonary surfactant

Pulmonary surfactant is essential for the maintenance of alveolar integrity and consists primarily of phospholipids, of which 80–85% is phosphatidylcholine (PC), with dipalmitoyl-PC (DPPC) or PC32:0 accounting for 40–60% and proteins ^10^. Anionic phospholipids [such as phosphatidylglycerol (PG), phosphatidylinositol (PI), phosphatidylethanolamine (PE), and sphingomyelin] account for the remainder. DPPC (PC32:0) is the primary essential PC molecule required for the surface reduction property of surfactant. Surfactant is synthesised and secreted by alveolar type II (AT-II) cells. Surfactant deficiency from impaired synthesis/secretion, increased breakdown (either by hydrolysis or oxidation), or inactivation/inhibition by biophysical inhibitors can lead to compromised alveolar surface tension ^11^. In mechanically ventilated patients, surfactant deficiency can exacerbate the initial lung pathology and impair lung compliance, thus worsening the pre-existing systemic hypoxaemia ^12^.

Lungs are the primary target for direct oxygen toxicity, and animal studies have consistently demonstrated that lungs exposed to high oxygen concentrations exhibited quantitative and qualitative alterations in surfactant composition and function ^13^. In in-vivo animal models, exposure to sub-lethal doses of oxygen results in decreased lung compliance, increased pulmonary leak, and inflammation with neutrophil migration. This is accompanied by hyaline membrane formation, alveolar septal oedema, fibrosis, and diffuse hyperplasia of alveolar epithelial cells ^14^. These changes mimic neonatal respiratory distress syndrome (nRDS) due to primary surfactant deficiency, implying that surfactant deficiency may contribute to acute hyperoxic lung injury.

Surfactant synthesis can be compromised due to hyperoxia. In isolated alveolar type-II cells, from rabbits exposed to hyperoxia, the surfactant phospholipid synthesis is compromised and there was a progressive development of acute lung injury ^15^. Moreover, exogenous surfactant replacement can significantly ameliorate hyperoxia-induced acute lung injury and improve alveolar phospholipid concentration ^16^. Besides the total surfactant pool size, hyperoxia can induce alterations in the surfactant composition with reductions in DPPC (PC32:0) and the PG/PI ratio, leading to compromised surfactant function ^17^. These *in-vitro* and *in-vivo* animal studies suggest that surfactant synthesis and function are significantly altered during hyperoxic conditions and likely contribute to adverse clinical outcomes; this concept has never been evaluated in humans.

### Oxidative stress and redox balance

The balance of oxidants and antioxidants in healthy physiological states is tightly regulated. Oxidative stress occurs when there is an imbalance of this equilibrium with increased oxidants. Alterations in this equilibrium can lead to a pro-inflammatory state with an influx of inflammatory cells, activation of cytokine cascades and increased vascular permeability ^18^. Hyperoxia related perturbations of this homeostatic balance results in the release of highly reactive mitochondrial mediators called reactive oxygen species (ROS) resulting in cellular damage ^19^. This imbalance also results in exhaustion of antioxidant mechanisms, exacerbating further tissue damage. Beyond ROS, other reactive nitrogen (RNS) and sulphur-based species (RSS) exist. The conceptual framework of the “reactive species interactome (RSI)” describes the complex chemical interaction between these reactive molecules and their downstream intracellular targets and metabolites contributing to organ dysfunction ^20^.

In hyperoxic states in critical illness, the balance between oxidative stress and endogenous antioxidants is altered, leading to a shift in extracellular redox status with a compromised ability to achieve whole-body redox balance and remove toxic molecules. This results in changes in the redox signalling and modulation of secondary messengers, causing mitochondrial dysfunction and cellular bioenergetic failure ^21^. Consequently, the main feature of hyperoxia demonstrated in *in-vivo* models and isolated cell cultures is cell death through apoptosis or necrosis ^22, 23^. Importantly, as redox status is an interconnected complex system, using single biomarkers from readouts of oxidative stress is challenging.

There remains a lack of understanding of oxygen related organ damage from in-vivo studies. Therefore, this mechanistic study (MecROX) will aim to address the mechanisms of hyperoxia, giving vital information for clinical management of mechanically ventilated patients requiring oxygen.

### Study hypotheses

**Figure 1.**
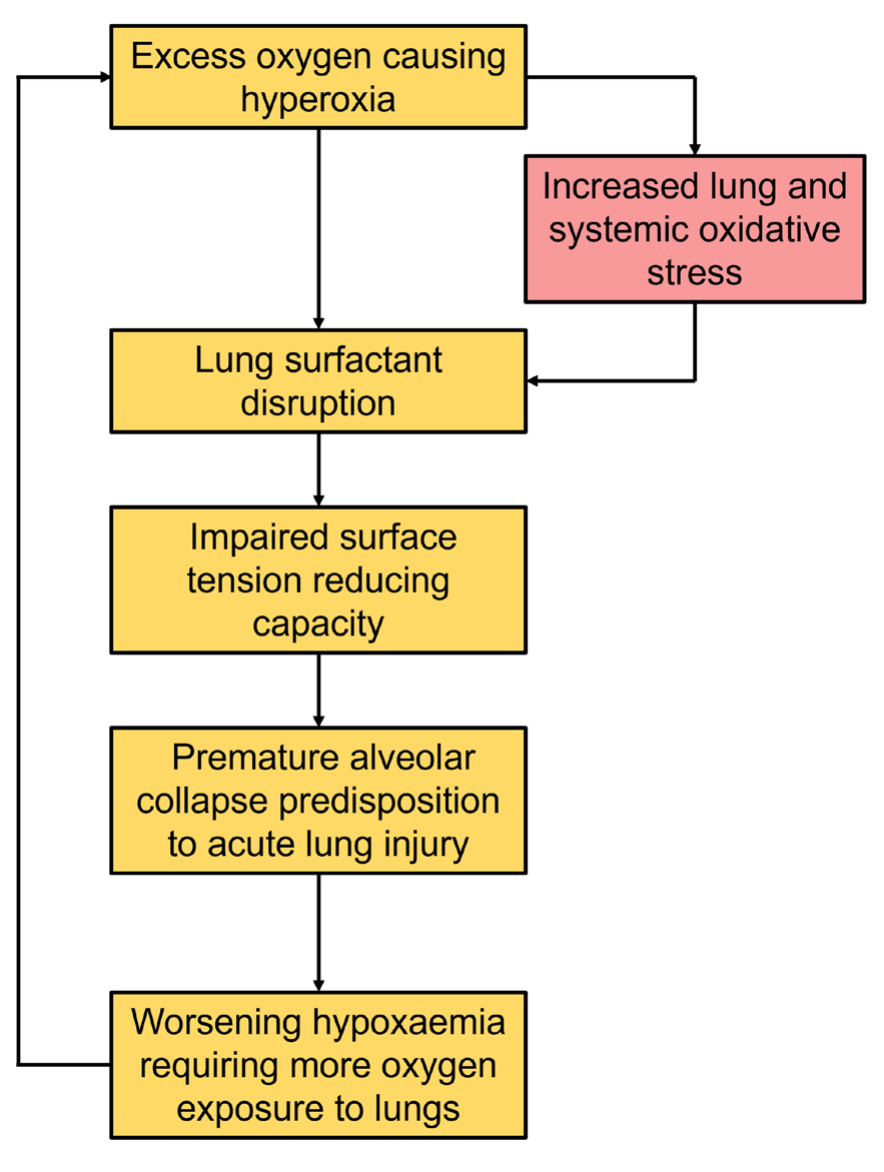
Pathophysiological process of the contribution of hyperoxia in the development of acute lung injury.

We hypothesise that hyperoxia may increase alveolar and systemic oxidative stress and adversely impact surfactant metabolism. Specifically, in mechanically ventilated patients: ( **i**) administration of high inspired oxygen concentrations will contribute to increased alveolar and systemic oxidative stress; **(ii)** increased alveolar and systemic oxidative stress will result in adverse changes in surfactant metabolism. We will characterise these metabolic phenotypes according to surfactant metabolism, alveolar and systemic oxidative stress. Stratification of these phenotypes may help to identify select groups that may benefit from targeted exogenous surfactant replacement, personalised therapeutic oxygen therapy and/or co-administration of targeted candidate therapeutic agents such as antioxidants to minimise surfactant inhibition and breakdown ( Figure 1).

### Aim and objectives

The primary aim of this study is to characterise in-depth the alveolar surfactant biology, oxidative stress and whole-body redox status in mechanically ventilated adult patients receiving two different oxygen therapy strategies.

### Study objectives

**Objective 1**: Quantify dynamic surfactant phospholipid composition, synthesis, and oxidative catabolism *in-vivo* and compare between the conservative and usual oxygen therapy group in mechanically ventilated patients.

**Objective 2**: Quantify lung and systemic oxidative stress and redox status by measuring the ‘reactive species interactome’ and compare between the conservative and usual oxygen therapy group mechanically ventilated patients.

**Objective 3**: Exploratory assessment of surfactant phenotype, lung and systemic oxidative stress and redox status, in relation to clinical correlates of oxygenation, ventilation, and clinical outcomes.

## Protocol

### Patient and Public Involvement

Patient and public are involved from the initial conception of the study. The PPI team concluded unanimously that this was an important research question. The study is also inclusive of a PPI member and a PPI coordinator. The PPI members contributed to the protocol development, grant application and design of the patient facing documents. The PPI team also suggested measures to improve recruitment. The PPI group will continue to meet twice yearly to assess the progress of the study and will contribute to the study dissemination after completion.

### Research design

**Figure 2.**
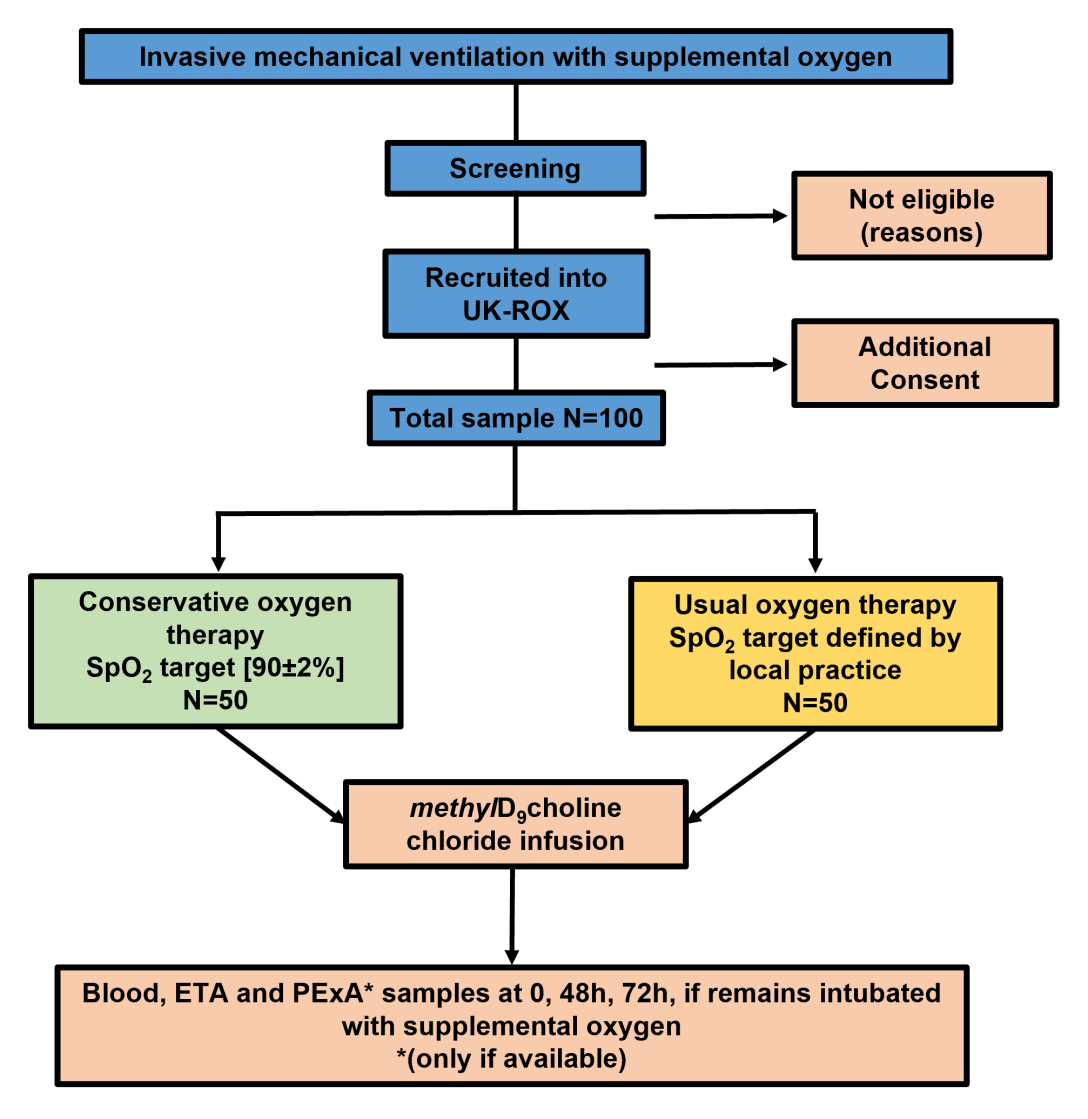
Study flowchart.

This is a prospective sub-study of the UK-ROX randomised controlled trial. The CONSORT flow diagram for the study is detailed below ( Figure 2). Participants form two centres (University Hospital Southampton and University Hospitals Plymouth) will be co-enrolled with the UK-ROX trial [1]. Following randomisation, patients will be assigned into either the conservative or standard oxygen therapy.

### Screening

Potential study participants of mechanically ventilated due to hypoxaemic respiratory failure, hospitalised patients aged ≥18 already enrolled into the UK-ROX study will be identified by the research team.

### Randomisation and UK-ROX interventions

All MecROX study participants will be already enrolled into the UK-ROX trial randomised to receive either conservative oxygen therapy (intervention) or usual oxygen therapy (control) using a central telephone or web-based randomisation service. Fifty patients from each group will be enrolled into the MecROX study. All interventions related to oxygen targets will comply with the UK-ROX protocol [1].

**Conservative oxygen target**: For the conservative group, the lowest concentration of oxygen will be administered to maintain the patient’s oxygen saturation (SpO _2_) at 90 ±2% (i.e., for patients receiving oxygen this should not rise above 92%).

**Usual oxygen target**: This is defined as local practice as determined by treating clinicians.

### Participants

All consecutive patients admitted will be screened according to the UK-ROX inclusion and exclusion criteria and they will be eligible if they fulfil the following criteria.

***Inclusion criteria***

•   Aged ≥ 18 years

•   Receiving invasive mechanical ventilation in the ICU for hypoxaemic respiratory failure

•   Receiving supplemental oxygen (fractional inspired concentration of oxygen (FiO _2_>0.21 at the time of enrolment)

•   Anticipated to be mechanically ventilated for minimum of 72 hours

***Exclusion criteria***

•   Previously randomised into the UK-ROX in the last 90 days

•   Currently receiving extra-corporeal membrane oxygenation (ECMO)

•   The treating clinician considers that one UK-ROX trial intervention arm is either indicated or contraindicated

### Consent process

Due to the nature of the UK-ROX trial, a deferred consent model has been adopted where eligible patients are randomised to receive the assigned treatment as soon as possible (no later than 12 hours after fulfilling the eligibility criteria). For MecROX, we will seek a patient informed deferred consent from a personal consultee opinion within 24–48 hours of UK-ROX randomisation. We anticipate that all patients enrolled into the study will lack the capacity to make decisions about their care or participation (commonly due to sedative medications and/or critical illness) at the time of enrolment. As a result, it will not be possible to discuss the study with the participant. If the participant does not have the capacity to provide informed consent, a personal consultee will be appointed, who may be a relative or close friend with whom to discuss the patient’s participation in the trial. After giving them the personal consultee information sheet, the research staff will seek the personal consultee’s opinion as to whether they think the patient would wish to take part in the study. If the personal consultee agrees that they believe the patient would want to participate, they will be asked to sign a personal consultee declaration form and a member of the research team will then countersign it. If no personal consultee is present or immediately available in person, we will seek an agreement via the telephone. This can occur during restrictions on visitations (in the case of COVID-19). If an agreement is obtained via the telephone, a member of the research team will complete the telephone personal consultee declaration form after giving information about the study from the personal consultee information sheet. When the participant regains the capacity to consent to the study after a personal or nominated consultee has agreed to their inclusion, a retrospective consent form will be completed and signed by the participant as above.

### Interventions

**Figure 3.**
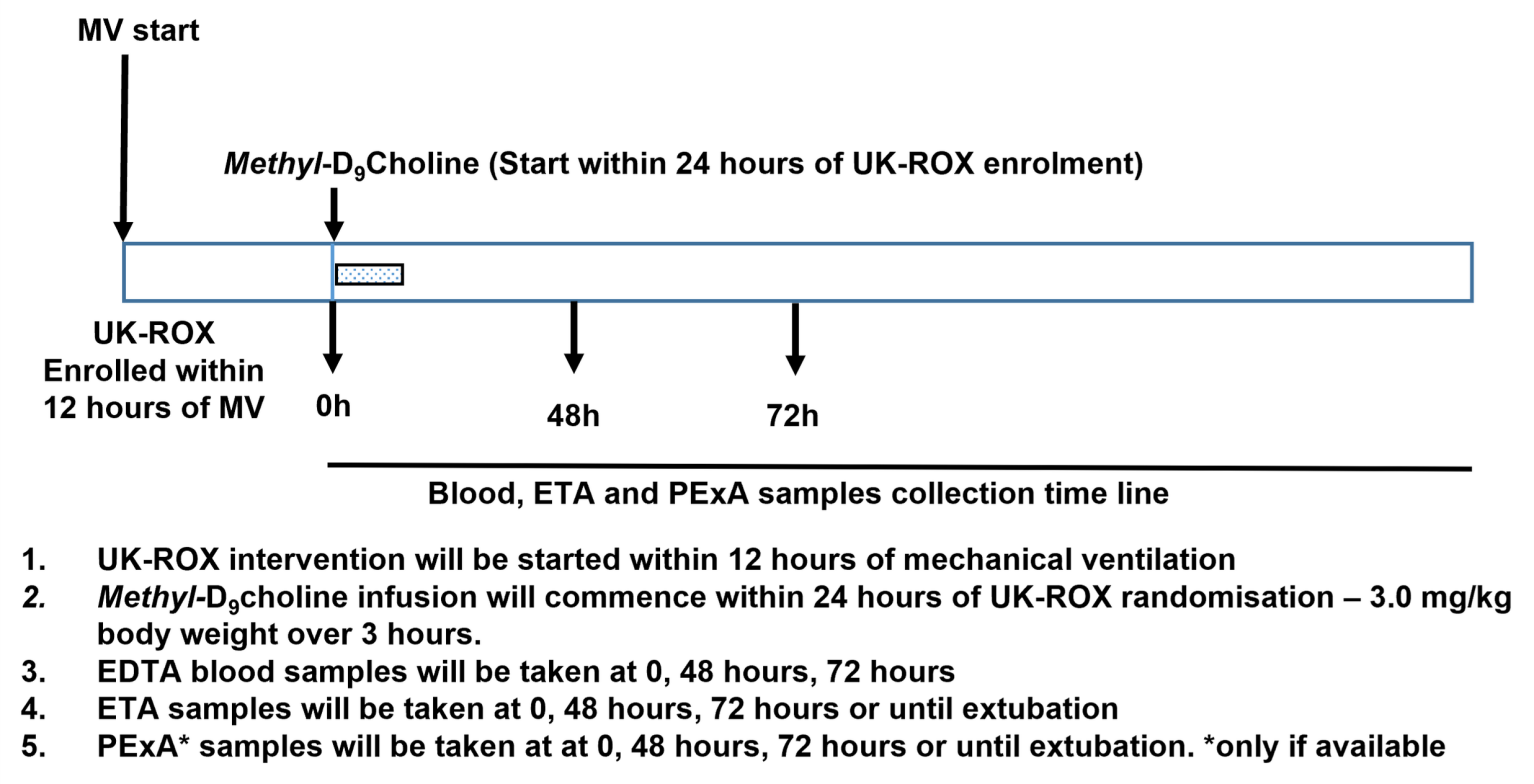
Sampling schedule and planned study interventions.

Study interventions and sampling schedule are presented in Figure 3.

***Methyl-D _9_ choline infusion***

**Figure 4.**
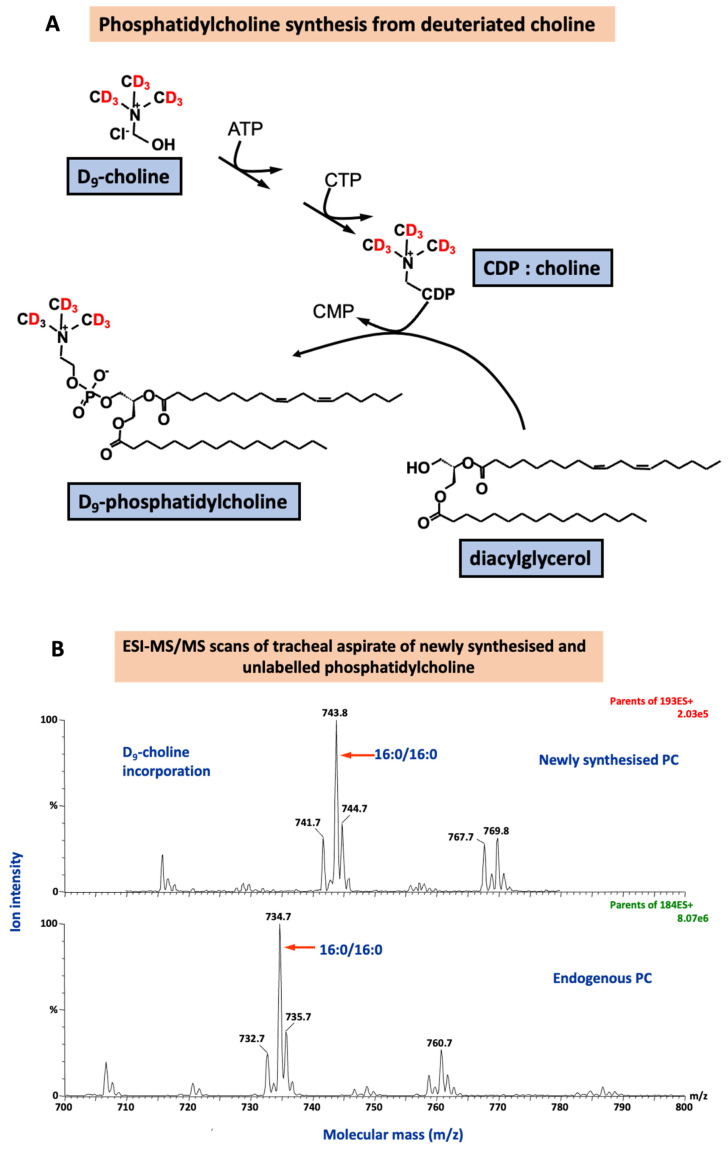
The incorporation of deuterated choline ( *methyl*-D _9_ choline chloride) to quantify surfactant phosphatidylcholine (PC) synthesis via the CDP-choline pathway ( **A**) and the corresponding mass spectra for endogenous PC composition and newly synthesised PC fraction. ATP: adenosine triphosphate; CDP: cytidine diphosphate; CMP: cytidine monophosphate; CTP: cytidine triphosphate.

Choline is an essential nutrient grouped within the vitamin B complex and is required as part of a normal healthy diet. Choline is a crucial component of the lung surfactant. Labelling naturally occurring choline ( *methyl-*D _9_ choline chloride) with a non-toxic, non-radioactive, stable isotope of hydrogen (deuterium, D) helps measure the rate of surfactant synthesis and breakdown in different diseases. This has been used successfully in several clinical studies of healthy adult volunteers and in adult and neonate patients with lung problems without any known side-effects ^24, 25^. *Methyl-*D _9_-choline chloride will be dissolved in water at 10 mg/ml and infused at a dose of 3 mg/kg body weight over 3 hours, will enable dynamic assessment of surfactant synthesis and turnover. *Methyl-*D _9_-choline incorporation into lung surfactant DPPC, the major surface-active component, will measure surfactant phospholipid synthesis ( Figure 4). It will answer the mechanistic question:

1. Does excess oxygen affect surfactant DPPC (PC32:0) synthesis and turnover?

A metabolic pathway for *methyl*-D _9_-choline incorporation into phospholipids is shown below, together with an example of the diagnostic mass spectrometry scans used for detection and quantification of unlabelled and deuterium-labelled PC.

We will aim to recruit patients and take the initial samples within 24 hours of randomisation into the UK-ROX study groups. The earliest time point will enable baseline assessment and subsequent 48 hours and 72 hours sampling times will assess the dynamic changes resulting from initiation of the intervention. The peak incorporation of *methyl-*D _9_ choline is between 48–72 hours after infusion, which will provide a measure of the maximal surfactant PC and LysoPC enrichment patterns monitoring both PC synthesis via the CDP choline pathway and the extent of enhanced breakdown through hydrolysis.

***Blood samples collection***

EDTA (10 ml) blood samples will be taken at baseline, 48, and 72 hours, (while hospitalised) after the *methyl-*D _9_-choline infusion. These samples will be taken only if the patient is still hospitalised. While in ICU, these samples are usually taken from pre-existing venous or arterial access lines. The collected blood samples will be stored at +4°C until transfer to the laboratory for processing. Processed samples taken at University Hospitals Plymouth will be transferred in frozen form to University Hospital Southampton for further analysis.

***Endotracheal tracheal aspirate***

Endotracheal aspirate (ETA) is a commonly performed procedure for secretion clearance in patients in the intensive care unit. Tracheal samples can be used to assess airway surfactant composition and metabolism. We have used tracheal aspirates to successfully isolate surfactant material in previous studies of healthy human volunteers and COVID-19 patients ^26^. Tracheal aspirate samples will be taken at 0, 48, and 72 hours, after the *methyl-*D _9_-choline infusion. The ETA samples will consist of blind suctioning by a cannula introduced through a port into the endotracheal tube. A volume of 20–50 ml saline will be administered, with an estimated recovery of 8–10 ml. This procedure will not interrupt the ventilator circuit and will not result in desaturation and is usually performed during physiotherapy sessions for mucus clearance. ETA samples will be taken into pre-labelled tissue culture (Falcon) tubes and stored at +4 ^°^C in the ICU sample fridge until transfer to the laboratory for processing.

***Particles of exhaled air (PExA)***

Small airway samples are the gold-standard for surfactant measurements. However, sampling from small airway is often very difficult as bronchoscopy and lavage is the only option. While safe and a routine procedure, bronchoscopy is an invasive method for airway sampling which require medical expertise and have potential for desaturations during the procedure. Moreover, repeated sampling will likely to require preoxygenation with high inspired oxygen which will interfere with trial interventions. To avoid this, PExA from patients will be measured by PExA device (Gothenberg, Sweden) ^27^. This will enable repeated small airway sampling for surfactant assessments without the use of bronchoscopy. This PExA device contains an optical particle counter (OPC) connected to an impactor for collection of samples at a diameter range of 0.41–4.55 µm. The PExA instrument will be connected to the ventilator outflow circuit to capture expiratory samples. The samples will be taken for an hour at 0, 48 and 72. The device will measure number of particles (count) and total accumulated mass (ng) of particles which will be collected onto a membrane for further biochemical analysis.

### Sample analysis

***Surfactant lipid composition and dynamic turnover***

Phospholipids, lysophospholipids and oxidised phospholipids molecular species will be analysed in tracheal fluid samples and from PExA. Mass spectrometric analysis of molecular species compositions of phosphatidylcholine (PC), phosphatidylglycerol (PG) and phosphatidylinositol (PI) in small volume ETA samples. Lysophosphatidylcholine products of PLA2-mediated hydrolysis of surfactant phospholipid will be determined by diagnostic precursor scans, together with molecular species compositions of intact surfactant phospholipid. Oxidation of unsaturated phospholipid initially generates higher mass peroxides, which then undergo chemical degradation to form truncated lower mass lipid aldehydes and hydroxyls. Intact and high and low mass oxidised phospholipids will be determined by specific MRM scans using electrospray ionization tandem mass spectrometry (ESI-MS/MS). An estimate of surfactant concentration will be determined by urea dilution analysis in parallel samples of bronchial fluid and plasma samples.

***Quantification of redox biology***

The extent of local and systemic oxidative stress with associated modifications of surfactant composition, alterations in cell signalling (due to interference with nitric oxide and hydrogen sulfide-related cell function) and shifts in redox status following the increased production of reactive oxygen species will be characterised in aliquots of pulmonary secretions and blood. Oxidative stress, nitric oxide, hydrogen sulfide and other redox related metabolites and products of ‘reactive species’ interactions will be quantified by an array of analytical platforms including ELISA, gas-phase chemiluminescence, HPLC, IC-MS, and LC-MS/MS to determine the following readouts: 8-isoprostanes, malondialdehyde (TBARS), nitrite, nitrate, total nitroso species, thiosulfate, sulfate, total free thiols, free and bound low-molecular weight thiols (including cysteine, homocysteine, glutathione), sulfide and polysulfide species according to local standard operating procedures.

### Outcome measures

***Primary outcome***

The difference of percentage of DPPC (PC32:0) in relation to total phosphatidylcholine composition (% of total PC in surfactant) at 48 hours between conservative and usual oxygen target groups.

***Secondary outcomes***

*1*. Surfactant index: This is a composite PC surfactant molecular index calculated from surfactant specific PC molecules (PC32:0, PC32:1 and PC30:0) and unsaturated surfactant PC34:1. This index will give a composite measure of surfactant PC alterations, which will provide a measure of surfactant PC status for the two different targets after 48 hours of oxygen therapy. *This outcome is a measure of surfactant specific PC composition.*

     Surfactant index = $\frac{\left\{PC32\text{:}0\text{+}PC32\text{:}1\text{+}PC30\text{:}0\right\}}{PC34\text{:}1}$

*2*. Surfactant phosphatidylcholine concentration (urea corrected) at 48 hours. *This outcome is a measure of endogenous surfactant level.*

*3*. Systemic oxidative stress: Total free thiols, lipid peroxides and total surfactant oxidation products *. This outcome will measure whole-body oxidative stress.*

***Secondary explanatory outcomes***

4. Surfactant total phosphatidylcholine and PC32:0 *methyl-*D _9_choline enrichment at 48 hours. *Measure of endogenous surfactant synthesis. This will measure the surfactant PC synthesis via the CDP-Choline pathway.*

*5*. Surfactant total lysoPC and lysoPC16:0 concentrations, composition and *methyl*-D _9_ choline enrichment at 48 hours. *This outcome is a measure of endogenous surfactant breakdown. This will help to assess dynamic surfactant PC breakdown through hydrolysis.*

*6*. Surfactant oxidised PC composition and concentrations at 48 hours. *Measure of endogenous surfactant breakdown. This will help to assess dynamic surfactant breakdown by oxidation.*

*7*. Whole- body redox balance by quantifying stable products of ROS (e.g., isoprostanes), RNS (e.g., nitrite, nitrate, nitrosation products) and RSS (e.g., total free thiols, thiosulfate, low molecular weight thiols including sulfide) at 48 hours from tracheal aspirates and plasma. *Measure of lung and systemic redox status*.

***Secondary exploratory outcomes***

8. Exploratory outcomes: Comparison of clinical outcomes (ICU mortality, hospital mortality, 90-day mortality, ICU, and hospital length of stay) in relation to surfactant abnormalities.

9. Exploratory outcomes: Comparison of clinical outcomes (ICU mortality, hospital mortality, 90-day mortality, ICU, and hospital length of stay) in relation to specific markers of oxidative stress.

### Sample size calculation

There is no previous index of surfactant damage documented in patients or animal models of acute hyperoxic lung injury. From ARDS and healthy volunteer studies, estimates of the mean (SD) for DPPC (PC32:0) composition are patients with ARDS 35.6% (SD 12.1%) and healthy controls 53.1% (SD 4.3%) ^10, 24^. Excess exposure will likely have alterations in DPPC composition from healthy volunteers but not significantly similar to the patients with ARDS, so we estimate that the standard deviation will be between 4.3 and 12.1 ( Table 1). With the higher estimate of SD, a sample size of 90 patients will achieve 90% power to detect a difference of 8.4% with a significance level of 0.05 using a two-sided two-sample t-test. Using the lower estimate, a sample size of 90 patients will achieve 90% power to detect a difference of 3.0% between groups with a significance level of 0.05 using a two-sided two-sample t-test. Allowing for an estimated 10% drop out we will recruit 100 patients.

**Table 1.** Surfactant DPPC compositions a from healthy volunteers and ARDS patients used for the power calculations.

Variable	Timepoint	Group	Mean (SD)	Minimum absolute difference which can be detected at 90% power, N=90
DPPC or PC32: 0	48	ARDS	35.6 (12.1)	8.4
	48	Healthy control	53.1 (4.3)	3.0

### Statistical analysis

The research hypothesis is that there is a difference in DPPC (PC32:0) fractional concentration between the conservative and usual oxygen groups. The primary endpoint is percentage DPPC (PC32:0) relative to total PC composition (% of total PC in surfactant) at 48 hours. For the primary analysis, we will use a multiple regression model adjusted for baseline to investigate the difference between the conservative and usual oxygen groups. If data are not normally distributed, we will investigate whether a log transformation improves normality. We will also perform an adjusted analysis to address clinical heterogeneity, using multiple regression, with up to 10 variables including the following baseline variables: age, gender, body mass index, percentage of PC32:0 in relation to total PC composition at admission, clinical conditions (e.g., sepsis, pneumonia) and concentration of inspired oxygen required at admission. We will also perform descriptive subgroup analysis for group differences and present them as box and whisker plots and scatter plots. As samples are collected from time points T=0, T=48 and T=72 hours as a secondary analysis, we will also investigate the use of mixed models to look at the difference between groups over time.

For the secondary outcomes of surfactant index; surfactant PC32:0, PC32:1, PC30:0; surfactant total phosphatidylcholine and PC32:0 *methyl-*D _9_choline enrichment; surfactant total lysoPC and lysoPC16:0 concentrations, composition and *methyl*-D _9_ choline enrichment and surfactant oxidised PC composition and concentrations (all measured at 48 hours), we will investigate the difference between groups using a two-sample t-test or Wilcoxon- Mann-Whitney test depending on the normality of data. If appropriate, adjusted analyses will also be performed as specified for the primary endpoint. The above will be repeated for the analysis of individual redox/oxidative stress markers listed above.

For the exploratory clinical outcomes of ICU mortality, hospital mortality and all mortality censored at 90 days, we will use Cox regression with time-varying covariates to investigate the effect of surfactant abnormalities and specific markers of oxidative stress on survival. For ICU and hospital length of stay, we will use cause-specific Cox regression model to account for the fact that patients may die before being discharged. As an exploratory analysis, we will illustrate the relationship between surfactant markers and oxidative stress markers using scatter plots and will quantify these relationships using regression models.

### Data management

The clinical data collection will be enhanced within the ICNARC CMP research platform. Additional data collection will be obtained beyond the standard data collection by the UK-ROX clinical trial. The additional data will include baseline demographic variables, ventilation, and oxygenation parameters. The principal investigator will oversee and be responsible for data collection, quality, and recording. The nested CMP research platform enables data collection to be incorporated within the ICNARC routine CMP data collection process, streamlining data linkage. Study data will be made available online at the time study results dissemination.

### Ethics approval and study management

The study is sponsored by the University Hospital Southampton NHS Foundation Trust. The study is approved by HRA and the London Bromley Research Ethics Committee, REC: 22/LO/0877 and IRAS: 320671. The study is registered on ISRCTN registry, ISRCTN61929838 ( https://doi.org/10.1186/ISRCTN61929838). The MecROX study is managed by the Southampton Clinical Trials Unit (CTU) and Sponsored by the University Hospital Southampton. Chief Investigator will ensure all study personnel are appropriately orientated and trained, oversee recruitment and report to the trial safety monitoring committee.

### Study dissemination

The findings of this study will be presented locally, nationally, and internationally in intensive care and respiratory conference and society meetings and published through peer review journals.

### Study status

As of 19 ^th^ of February 2024, both sites are open for recruitment and recruited 25 participants so far.

### Strengths of the study

This is the first study to investigate the lung surfactant metabolism and systemic redox markers from mechanically ventilated patients with different oxygen targets providing a mechanistic in-vivo evaluation of oxidative stress and redox biology in critical illness. Results will inform underlying mechanisms of hyperoxia and hyperoxemia.

## Discussion

MecROX is a mechanistic study that aims to determine the mechanisms underlying hyperoxia and hyperoxemia induced lung surfactant and whole-body redox changes in-vivo. While oxygen therapy is essential for critically ill mechanically ventilated patients, there are potentially many harmful effects from hyperoxia, as demonstrated by several studies of hospitalised patients with acute conditions. Oxygen toxicity due to hyperoxia/hyperoxaemia is a well-recognised phenomenon in animal studies. Hyperoxaemia may exacerbate whole-body oxidative stress leading to cell death, autophagy, mitochondrial dysfunction, biogenetic failure with eventual multi-organ failure resulting in poor clinical outcomes. However, in-vivo human models evaluating hyperoxia-induced organ damage in mechanically ventilated patients are lacking. Decreased surfactant synthesis due to cellular apoptosis and increased surfactant breakdown by oxidation/hydrolysis can result in surfactant deficiency during states of increased oxidative stress. However, the influence of hyperoxia on direct surfactant damage and whole-body redox status has never been evaluated before in relation to various oxygen targets. It is critical to assess the alveolar environment to quantify the oxidative stress and redox imbalance to minimise surfactant breakdown to prevent worsening lung atelectasis and hypoxaemia.

This proposed study will mechanistically evaluate the in-vivo surfactant metabolism using a novel stable isotope technique and various platforms of mass spectrometry analytical methods. Surfactant phospholipid breakdown through hydrolysis (lysophospholipids) and oxidation (oxidised phospholipids) and redox disequilibrium (‘reactive species interactome’) will be quantified from sequential plasma and bronchial samples using an array of analytical platforms. These assessments will help characterise the alveolar and systemic redox status during different oxygen targets to stratify patients according to evidence of oxidative surfactant damage that may be responsive to targeted administration of therapies to minimise oxidative stress in the future.

## Conclusions

This observational study aims to characterise in-depth the molecular mechanisms relating to oxygen therapy in mechanically ventilated patients enrolled in the UK-ROX clinical trial receiving oxygen therapy to two different target targets. The study will collect serial biological samples to evaluate dynamic surfactant phospholipid concentrations, composition, synthesis, catabolism, and global assessment of whole-body oxidative stress and redox status (markers of the “reactive species interactome”). By doing so, the study aims to provide a mechanistic link between pathophysiology and clinical outcomes in critically ill intensive care patients receiving oxygen therapy.

## Data Availability

No data are associated with this article.
