# Supplementary material for: Oxidative stress, redox status and surfactant metabolism in mechanically ventilated patients receiving different approaches to oxygen therapy (MecROX): An observational study protocol for mechanistic evaluation
Source: NIHR Open Res. 2024 Jul 8;4:23. Originally published 2024 Apr 26. [Version 2] doi: 10.3310/nihropenres.13567.2 (PMC11320187; doi:10.3310/nihropenres.13567.2)
Supplement: Peer review history

# Reviewer report: Reviewer response for version 2

**DOI:** 10.3310/nihropenres.14842.r32272

**Published:** 2024-07-12

**Authors:** Adam Boulton, Joyce Yeung

**Affiliations:** 1 University of Warwick Warwick Clinical Trials Unit (Ringgold ID: 572395), Coventry, England, UK

We thank the authors for responding to our comments and making appropriate revisions. We have no further critical comments.

Is the study design appropriate for the research question?

Yes

Is the rationale for, and objectives of, the study clearly described?

Yes

Are sufficient details of the methods provided to allow replication by others?

Yes

Are the datasets clearly presented in a useable and accessible format?

Not applicable

Reviewer Expertise:

Peri-operative care, critical care, resuscitation.

We confirm that we have read this submission and believe that we have an appropriate level of expertise to confirm that it is of an acceptable scientific standard.

# Reviewer report: Reviewer response for version 2

**DOI:** 10.3310/nihropenres.14842.r32271

**Published:** 2024-07-09

**Authors:** Noah H Hillman

**Affiliations:** 1 Cardinal Glennon Children's Hospital, Saint Louis University School of Medicine, Saint Louis, USA

The additional information provided by the authors help clarify the methods of the study.

Is the study design appropriate for the research question?

Yes

Is the rationale for, and objectives of, the study clearly described?

Yes

Are sufficient details of the methods provided to allow replication by others?

Yes

Are the datasets clearly presented in a useable and accessible format?

Not applicable

Reviewer Expertise:

Surfactant function

I confirm that I have read this submission and believe that I have an appropriate level of expertise to confirm that it is of an acceptable scientific standard.

# Reviewer report: Reviewer response for version 1

**DOI:** 10.3310/nihropenres.14727.r31753

**Published:** 2024-06-28

**Authors:** Adam Boulton, Joyce Yeung

**Affiliations:** 1 University of Warwick Warwick Clinical Trials Unit (Ringgold ID: 572395), Coventry, England, UK

This paper is a study protocol for a mechanistic sub-study of the UK-ROX trial. This mechanistic evaluation aims to provide a scientific rationale and mechanistic explanation for the clinical findings of UK-ROX, specifically focussing on lung surfactant composition, and alveolar and systemic oxidative stress/redox status. In review of this manuscript, we have referred to some standard items in Standard Protocol Items for Observational Studies (SPIROS).

 The paper is generally well-written. It is structured appropriately and follows a logical progression. Explanations are clear and in appropriate detail. The background is described in sufficient detail, whilst also being accessible to the non-expert reader; allowing understanding of the rationale behind this study. Figure 1 is a good example of this. 

 The hypothesis and rationale for chosen outcomes were well-justified and detailed. We are reassured by the expertise within the very experienced named authorship team. This research group in Southampton has well-regarded expertise within this area and appears to be the ideal site/group to undertake this work. 

 We think it would be valuable for the authors to expand on the following points.

 The paper would benefit from further elaboration of the patient and public involvement. Whilst it is clear there was PPI, I am unclear how this has contributed to and shaped the study. Can the authors describe more specifically how the PPI led to changes in protocol development and design of the patient facing documents? Can the authors provide a rationale for meeting with the PPI group twice yearly and how this may influence the study? Similarly, it would be helpful for readers if the PPI group was briefly described. 

 A short paragraph of study strengths is reported. Would the paper benefit from a similar paragraph detailing potential limitations? 

 Are there any additional potential harms to participants that take part in this mechanistic sub-study?

 Can the authors clarify whether consent procedures cover storage of biological specimens and or analyses in the future?

 Can the authors add any funding information?

 Are there any plans to give access to full protocol or participant-level data? What is the data sharing policy?

Is the study design appropriate for the research question?

Yes

Is the rationale for, and objectives of, the study clearly described?

Yes

Are sufficient details of the methods provided to allow replication by others?

Yes

Are the datasets clearly presented in a useable and accessible format?

Not applicable

Reviewer Expertise:

Peri-operative care, critical care, resuscitation.

We confirm that we have read this submission and believe that we have an appropriate level of expertise to confirm that it is of an acceptable scientific standard, however we have significant reservations, as outlined above.

# Response

**Published:** 2024-07-02

**Authors:** Ahilanandan Dushianthan

**Affiliations:** General Intensive Care Unit, University Hospital Southampton, Southampton, Hampshire, UK

Thank you for the reviewer's comments. Please see our responses to the comments below. 

**1.** The paper would benefit from further elaboration of the patient and public involvement. Whilst it is clear there was PPI, I am unclear how this has contributed to and shaped the study. Can the authors describe more specifically how the PPI led to changes in protocol development and design of the patient facing documents? Can the authors provide a rationale for meeting with the PPI group twice yearly and how this may influence the study? Similarly, it would be helpful for readers if the PPI group was briefly described.

**Response**: We have modified this paragraph to provide a detailed explanation of the PPI activities. 

**2**. A short paragraph of study strengths is reported. Would the paper benefit from a similar paragraph detailing potential limitations? 

**Response: **This is a protocol publication, any limitations (e.g., recruitment, analysis, etc.) of the study will be detailed once the study is complete.

**3.** Are there any additional potential harms to participants that take part in this mechanistic sub-study?

**Response:** We have added a new paragraph in the intervention section to explain study procedures and risk mitigation. 

**4. **Can the authors clarify whether consent procedures cover storage of biological specimens and or analyses in the future?

**Response:** We have introduced a new sentence in the consent process section to include consent for the storage of samples for future use.

**5. **Can the authors add any funding information?

**Response:** This is already provided in the box before the introduction as;

 Grant information: This project is funded by the National Institute for Health and Care Research (NIHR) under its Efficacy and Mechanism Evaluation (EME) programme (NIHR151287) and supported by the University Hospital Southampton NIHR Biomedical Research Centre (BRC). The views expressed are those of the author(s) and not necessarily those of the NIHR, NHS or the Department of Health and Social Care.

*The funders had no role in study design, data collection and analysis, decision to publish, or preparation of the manuscript.*

**6. **Are there any plans to give access to full protocol or participant-level data? What is the data sharing policy?

**Response: **We have introduced additional sentences to include data availability after study completion and full protocol availability.

# Reviewer report: Reviewer response for version 1

**DOI:** 10.3310/nihropenres.14727.r31752

**Published:** 2024-05-25

**Authors:** Noah H Hillman

**Affiliations:** 1 Cardinal Glennon Children's Hospital, Saint Louis University School of Medicine, Saint Louis, USA

Overall the manuscript describes the study design and purpose for the study. MecROX is a sub-study within the larger UK-ROX trial. It is designed to look at the effects of oxidative stress on surfactant metabolism utilizing a stable isotope of choline. The justification for the deferred consent process is described and has been vetted with patient panels. 

 The introduction provides strong evidence and rationale for the hypothesis from animal models. 

 The flow charts demonstrate both eligibility requirements and the timing of the samples collected. 

 The description of the assays is appropriate for people to understand the types of oxidative stress markers to be evaluate in blood and tracheal aspirates. 

 Power calculation is appropriate for the study design and the statistical methods are appropriate. 

 Other than the arrow on Figure 2 for additional consent possibly being pointed in the opposite direction, the figures are informative to the reader.

 Overall the manuscript is well written and will allow researcher and the public to learn about this important substudy

Is the study design appropriate for the research question?

Yes

Is the rationale for, and objectives of, the study clearly described?

Yes

Are sufficient details of the methods provided to allow replication by others?

Yes

Are the datasets clearly presented in a useable and accessible format?

Not applicable

Reviewer Expertise:

Surfactant function

I confirm that I have read this submission and believe that I have an appropriate level of expertise to confirm that it is of an acceptable scientific standard.
